# Changes of serum MMP-9, NSE, MPO levels and prognostic influencing factors in patients with intracranial aneurysm undergoing interventional embolization at different treatment timing

**DOI:** 10.5937/jomb0-44364

**Published:** 2024-01-25

**Authors:** Chunmiao Wu, Xingyu Dong, Qiang Li, Shengming Liu, Yuhao He, Yang Zhang, Sunfu Zhang

**Affiliations:** 1 Chengdu Third Peopležs Hospital, Department of Neurosurgery, Chengdu, Sichuan, China

**Keywords:** intracranial aneurysm, interventional embolization, matrix metalloproteinase-9, neuroenolase, myeloperoxidase, intrakranijalna aneurizma, interventna embolizacija, matriks metaloproteinaza-9, meuroenolaza, mijeloperoksidaza

## Abstract

**Background:**

To analyzes the changes in serum levels of matrix metalloproteinase-9 (MMP-9), neuroenolase (NSE), myeloperoxidase (MPO) and prognostic factors in patients with intracranial aneurysm (IA) undergoing interventional embolization at different treatment times.

**Methods:**

A retrospective analysis was made of 200 IA patients admitted to our department from January 2018 to June 2021 was performed. All patients underwent interventional embolization. According to the timing of surgery, the patients were divided into an early group (n=120, onset to surgery ≤72 h) and a delayed group (n=80, onset to surgery >72 h). The effect of embolization, complications and neurological deficit scale (NDS) scores were compared between the two groups. Serum MMP-9, NSE and MPO levels were compared before and after surgery, and the prognosis of all patients within 2 years after surgery was assessed by the Glasgow outcome scale (GOS) and divided accordingly into the good prognosis group (n=147) and the poor prognosis group (n=53) accordingly, and the prognostic factors influencing the patients were analyzed univariately and multifactorially.

## Abstract (sr)

**Uvod:**

Cilj je bio da se analiziraju promene u serumskim nivoima matriks metaloproteinaze-9 (MMP-9), neuroenolaze (NSE), mijeloperoksidaze (MPO) i prognostičkih faktora kod pacijenata sa intrakranijalnom aneurizmom (IA) koji su podvrgnuti interventnoj embolizaciji u različitim vremenima lečenja.

**Metode:**

Urađena je retrospektivna analiza 200 pacijenata sa IA koji su primljeni na naše odeljenje od januara 2018. do juna 2021. godine. Svi pacijenti su podvrgnuti interventnoj embolizaciji. Prema vremenu operacije, pacijenti su podeljeni u ranu grupu (n=120, početak operacije ≤72 h) i odloženu grupu (n=80, početak operacije >72 h). Efekat embolizacije, komplikacije i rezultati skale neurološkog deficita (NDS) su upoređeni između dve grupe. Nivoi MMP-9, NSE i MPO u serumu su upoređeni pre i posle operacije, a prognoza svih pacijenata u roku od 2 godine nakon operacije procenjena je Glasgov skalom ishoda (GOS) i prema tome podeljena u grupu sa dobrom prognozom (n=147) i grupa sa lošom prognozom (n=53) shodno tome, a prognostički faktori koji utiču na pacijente analizirani su univarijantno i multifaktorski.

**Rezultati:**

Nakon operacije, stopa potpune embolije bila je veća u ranoj grupi nego u odloženoj grupi (P<0,05). Nakon operacije, nije bilo statističke značajnosti u poređenju pojedinačnih stopa komplikacija u obe grupe (P>0,05). U 3d, 1 mesec i 6 meseci nakon operacije, rezultati NDS pacijenata u obe grupe su bili niži od onih pre operacije, a poređenje NDS rezultata pacijenata u obe grupe u različitim vremenskim tačkama je bilo statistički značajno ( P<0,05). Posle operacije, nivoi MMP-9, NSE i MPO u serumu su bili niži u obe grupe nego pre operacije, a bili su niži u ranoj grupi nego u odloženoj grupi (P<0,05). Rezultati GOS-a su pokazali da je u roku od 2 godine nakon operacije bilo 97 i 23 slučaja sa dobrom i lošom prognozom u ranoj grupi i 54 i 26 slučajeva sa dobrom i lošom prognozom u odloženoj grupi, respektivno, a stopa dobre prognoze u ranoj grupi grupa je bila veća od one u odloženoj grupi (P<0,05). Multifaktorska analiza je pokazala da su odložena operacija, aneurizma u zadnjoj cirkulaciji, širina vrata aneurizme >4,5 mm, stepen III-IV po Fisheru, stepen III-IV Hunt-Hess i hipertenzija nezavisni faktori rizika za lošu prognozu nakon interventne embolizacije IA (P <0,05).

**Zaključak:**

Rana interventna embolizacija kod pacijenata sa IA može poboljšati njihovu potpunu stopu embolizacije i smanjiti nivoe MMP-9, NSE i MPO u serumu; odložena operacija, aneurizma u zadnjoj cirkulaciji, širina vrata aneurizme >4,5 mm, Fisher stepen III-IV, Hunt-Hess stepen III-IV i hipertenzija su snažno povezani sa lošom prognozom nakon interventne embolizacije kod pacijenata sa IA.

## Introduction

Intracranial aneurysms (IA) occur in the congenitally dysplastic part of the intracranial arterial wall. Depending on the location of the aneurysm, they are classified as anterior cerebral aneurysms, middle cerebral aneurysms, anterior communicating aneurysms, posterior communicating aneurysms, and vertebrobasilar aneurysms. The etiology of the disease is unknown and related to many factors, such as congenital arterial wall defects [1], hemodynamic alterations [2], atherosclerosis [3], and vasculitis [4]. The main clinical symptoms include nausea and vomiting, severe headache, meningeal irritation and altered consciousness. IA rupture is the main cause of subarachnoid hemorrhage and can also lead to serious complications, such as hydrocephalus, vasospasm or ischemic stroke, resulting in a poor prognosis for patients [5] [6]. Traditional craniotomy has been performed before. It can clear the blood in the fundus cistern and prevent cerebral vasospasm, but this kind of surgery is invasive and causes huge damage to the skull. At present, Endovascular embolization (EVET) is the main treatment for IA. Compared with traditional craniotomy, intravascular embolization has the advantages of being less invasive, less disabling and less lethal, and less postoperative complications, which is of great importance to improve the condition of these patients. However, due to the differences of individual patient, in some cases, the timing of surgery may vary due to the underlying diseases of comorbidity and other reasons, and the impact of different surgical timing on the outcome of patients may vary. Sonig et al. [7] concluded that early diagnosis and treatment can improve the outcome of EVET treatment and improve the surgical prognosis. However, some scholars [8] believe that if surgery is delayed, patients' vasospasm and brain tissue swelling will improve, and the resorption and anatomical level of blood clots will become clear, thus reducing the risk and difficulty of surgery. There is still some clinical controversy about the timing of EVET surgery. In addition, studies [9] have shown that the prognosis of EVET treatment may be affected by many factors, but the specific factors still need to be further clarified. Matrix metalloproteinase-9 (MMP-9) is a calcium-zinc-dependent proteolytic enzyme that degrades almost all components of the extracellular matrix, is involved in the body's inflammatory response, and can accumulate in large amounts around aneurysms [10]. Neuroenolase (NSE) is mainly found in nerve and endocrine tissues; when nerve tissue is damaged, its level in the circulation increases, which is an important indicator of the degree of brain tissue damage [11]. Myeloperoxidase (MPO) is an oxidase produced by activated neutrophils, monocytes and macro phages. Its serum expression level is associated with the degree of injury in patients with cerebrovascular disease [12]. On this basis, this study analyzed the changes in serum MMP-9, NSE, and MPO levels and their prognostic factors in IA patients receiving EVET at different treatment times, to provide a reference for selecting of surgery and obtaining prognostic risk factors in IA patients receiving EVET treatment.

## Materials and methods

### Patients & subgroups

Retrospective analysis of 200 patients with IA admitted to our department from January 2018 to June 2021, all of whom were treated with interventional embolization. Patients were divided into an early group (n=120, onset to surgery ≤72 h) and a delayed group (n=80, onset to surgery >72 h) according to the timing of This study was performed after approval by the ethics committee of our hospital.

### Inclusion criteria 

(1) Fulfil the diagnostic criteria for IA [13]: Clinical manifestations included headache, impaired consciousness, actinic nerve palsy and increased intracranial pressure. Localized vessel wall damage with abnormal bulging was seen on cranial CT, digital subtraction angiography (DSA) and 3D-CT angiography, which confirmed intracranial aneurysm and visible intracranial hematoma. (2) All patients underwent interventional embolectomy at our hospital. (3) They were informed about the research situation and signed the relevant agreement. (4) Baseline information was complete and they actively cooperated with all efforts. (5) Prognostic outcome was followed up.

### Exclusion criteria 

(1) Recurrent aneurysm. (2) Complicated with intracranial space occupying lesions. (3) Intracranial hematoma caused by trauma. (4) Combined with moyamoya disease, arteriovenous malformation and other types of cerebrovascular disease. (5) History of craniocerebral surgery. (6) Contraindications to surgery exist [14]. (7) There is a serious infection or malignant tumor. (8) EVET or craniotomy clipping has been performed in other hospitals.

### Surgical regimen

Both groups were treated with interventional embolization. The early group was performed within 72 h of onset and the delayed group was performed after 72 h of onset.

Preoperative preparation: Aspirin enteric-coated tablets (Shanghai Baolong Pharmaceutical Co., Ltd., H31022886, specification: 25 mg) 200 mg and clopido grel sulfate tablets (Hunan Dino Pharmaceutical Co., Ltd., H20203609, specification: 75 mg (based on C16H16ClNO2S)) 150 mg were given to both groups 1h before surgery, in a single dose.

Intraoperative procedure: general anesthesia with tracheal intubation, supine position, placement of a 6F arterial sheath after conventional puncture of the right femoral artery, DSA examination to confirm the morphology, location, size and cerebrovascular status of the aneurysm in each patient, and selection of the appropriate diameter spring coil accordingly. After DSA, the whole process of heparinisation was performed to prevent intra-arterial thrombosis. The best working angle was chosen. The aneurysms were tightly packed with spring coils using the Russian doll technique. After the aneurysm cavity had disappeared, the tubes were removed and the groin was locally pressurized and banded. 

Postoperative treatment: Postoperative subcutaneous injection of low molecular weight heparin calcium injection (Hebei Changshan Biochemical Pharmaceutical Co., Ltd., H20063910, specification: 0.4 mL: 4100AXaIU) 5000 U, 2 times/d, 5 d continuous treatment. Tirofiban hydrochloride for injection (Shenyang Xinma Pharmaceutical Co., Ltd., H20153204, Specification: 5 mg (as tirofiban)) was adminis tered at 4 mL/h and intravenous micro pump was discontinued until the 1st postoperative day or 3h after oral administration of other drugs. Oral aspirin enteric-coated tablets 100 mg once/d and clopidogrel sulfate tablets 75 mg once/d from the 1st postoperative day for 3 months.

### Clinical data collection

Clinical data were collected from both groups, including age, gender, timing of surgery, aneurysm location, aneurysm diameter, aneurysm neck width, Fisher grade, Hunt-Hess grade, hypertension, diabetes, hyperlipidemia, and smoking history and so on. 

(1) Embolic effect: The DSA examination results were used to determine the degree of postoperative arterial embolization in both groups, i.e., partial embolization if the embolization area did not exceed 90%, basic embolization if the embolization area was 91%-99%, and complete embolization if the embolization area was 100% [7].

(2) Complications: Postoperative hydrocephalus, cerebral vasospasm, postoperative rebreeding, limb dysfunction, consciousness disorders, and electrolyte disturbances, etc. were mainly recorded in both groups.

(3) Neurological deficit scale (NDS) scores: The assessment scale was a rating standard for the degree of clinical neurologic deficits in stroke patients (1995), assessments were performed preoperatively, 3d postoperatively, 1 month postoperatively, and 6 months postoperatively, all by the same neurosurgeon. The assessment included 8 items such as consciousness and speech. The total score of 0-15 is mild neurological deficit, 16-30 is moderate neurological deficit, and 31-45 is severe neurological deficit.

(4) Serum MMP-9, NSE and MPO levels: The detection time was preoperatively and 1d postoperatively, 4 mL of peripheral venous blood was drawn, centrifuged at 3500 r/min for 10 min, and 0.5 mL of supernatant was frozen and stored. The levels of MMP-9, NSE and MPO in patients' serum were effectively detected by ELISA, and the standard curve was drawn with the optical density value as the ordinate and the standard concentration as the abscissa. According to the optical density value of serum samples, the concentration can be found out on the standard curve. The concentration of the specimen= the concentration found on the standard curve. The kits were purchased from Shanghai Yubo Biotechnology Co. (Shanghai, China).

### Prognostic evaluation & subgroups

The Glasgow outcome scale (GOS) scores among patients followed up at 2 years after surgery were 1 for death, 2 for vegetative survival, 3 for severe disability, 4 for mild disability, and 5 for good recovery. And accordingly, all patients were divided into the good prognosis group (4-5 points) and the poor prognosis group (1-3 points).

### Statistical analysis

The data were processed by Statistical Product and Service Solutions (SPSS) 22.0 software (IBM, Armonk, NY, USA) and the figures were drawn by GraphPad Prism 8.0 software (La Jolla, CA, USA). Count data are expressed as percentage (%) and analysis using Fisher's test. Measurement data are expressed as mean ± standard deviation (x ± s), compared using ANOVA with repeated measures design and t test using pairwise comparisons. A multivariate logistic regression model analyzed the prognostic factors. P<0.05 for statistical significance.

## Results

### Embolization effect

After surgery, the complete embolism rate was higher in the early group than in the delayed group (88.33% vs 75.00%, P<0.05) (Table 1).

**Table 1:** Embolization effect n, %. Note: Analysis using χ^2^ test.

Groups	Partial embolism	Basic embolism	Complete embolism
Early Group<br>(n=120)	0 (0.00)	14 (11.67)	106 (88.33)
Delayed group<br>(n=80)	0 (0.00)	20 (25.00)	60 (75.00)
χ^2^	-	-	6.048
P	-	-	0.014

### Complications

After surgery, 2 cases of postoperative rebleeding and 2 cases of electrolyte disturbance occurred in the early group; 1 case of hydrocephalus, 1 case of postoperative rebleeding, 2 cases of limb dysfunction, 2 cases of consciousness disorder, and 1 case of electrolyte disturbance occurred in the delayed group, and there was no statistical significance in the comparison of each complication rate in both groups (P>0.05).

### NDS score

**Figure 1:**
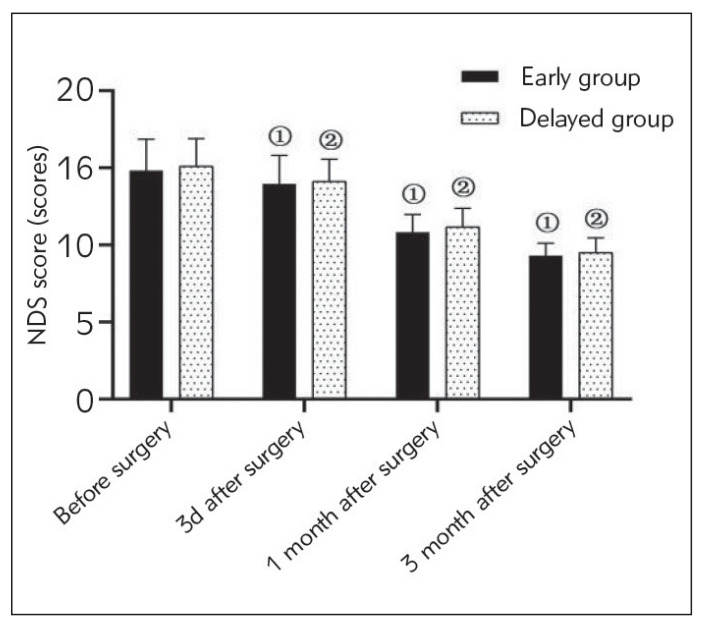
NDS score (scores). Note: Analysis using ANOVA with repeated measures design. As compared with the early group before surgery, (1) (13.96±1.84)/ (10.83±1.15)/(9.30±0.80) vs (14.82±2.03), t=3.439/18.734/27.713, P<0.001; As compared with the delayed group before surgery, (2) (14.13±1.42)/(11.17±1.20)/(9.50±0.95) vs (15.11±1.79), t=3.836/16.352/24.761, P<0.001. NDS: Eurological deficit scale.

There was no statistically significant comparison of NDS scores in both groups before and 3 d, 1 month, and 6 months after surgery (P>0.05). At 3 d, 1 month, and 6 months after surgery, the NDS scores of patients in both groups were lower than those before surgery, and the intra-group comparison of NDS scores of patients in both groups at different time points was statistically significant (P<0.05) (Figure 1).

### Serum MMP-9, NSE, MPO levels

**Figure 2:**
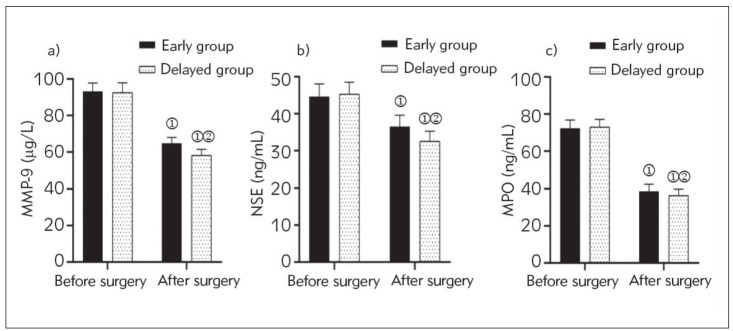
Serum MMP-9, NSE and MPO levels. (a) Serum MMP-9 levels (μg/L); Note: Analysis using ANOVA with repeated measures design. As compared with the same group before surgery, (1) (93.21±4.57) vs (64.76±3.18), t=55.977, P<0.001; (92.52±5.37) vs (58, 23±3.26), t=48.821, P<0.001; as compared with the early group after surgery, (2) (64.76±3.18) vs (58.23±3.26), t=14.084, P<0.001. MMP-9: matrix metalloproteinase-9. (b) Serum NSE levels (ng/mL); Note: Analysis using ANOVA with repeated measures design. As compared with the same group before surgery, (1) (44.63±3.42) vs (36.52±3.10), t=19.247, P<0.001; (45.30±3.22) vs (32.56±2.69), t=27.158, P<0.001; As compared with the early group after surgery, (2) (36.52±3.10) vs (32.56±2.69), t=9.321, P<0.001. NSE: neuroenolase. (c) Serum MPO levels (ng/mL). Note: Analysis using ANOVA with repeated measures design. As compared with the same group before surgery, (1) (72.41±4.42) vs (38.65±3.83),t=63.233, P<0.001; (73.06±4.08) vs (36.34±3.47), t=61.320, P<0.001; as compared with the early group after surgery, (2) (38.65±3.83) vs (36.34±3.47), t=4.336, P<0.001. MPO: myeloperoxidase.

After surgery, the serum MMP-9, NSE, and MPO levels were lower than those before surgery in both groups, and they were lower in the early group than in the delayed group (P<0.05) (Figure 2).

### Prognosis

GOS results showed that within 2 years after surgery, there were 97 and 23 cases with good and poor prognosis in the early group and 54 and 26 cases with good and poor prognosis in the delayed group, respectively, and the good prognosis rate in the early group was higher than that in the delayed group (80.83% vs 67.50%, P<0.05) (Table 2).

**Table 2:** Prognosis n, %. Note: Analysis using χ^2^ test. GOS: Glasgow outcome scale.

Groups	GOS score					Good prognosis
	1 score	2 scores	3 scores	4 scores	5 scores	
Early Group<br>(n=120)	0	6	17	57	40	97 (80.83)
Delayed group<br>(n=80)	0	10	16	37	17	54 (67.50)
χ^2^	-	-	-	-	-	4.613
P	-	-	-	-	-	0.032

### Univariate analysis of prognostic influencing factors

Univariate analysis showed that poor prognosis after IA interventional embolization was related to patients' surgery timing, aneurysm location, ane urysm neck width, Fisher grade, Hunt-Hess grade, and hypertension (P<0.05). It was not related to patients' age, gender, aneurysm diameter, diabetes, hyperlipidemia, or smoking history (P> 0.05) (Table 3).

**Table 3:** Univariate analysis of prognostic influencing factors (n, %). Note: Analysis using χ^2^ test. ^(1)^Fisher grade: Grade I: CT showed no hematocele. Grade II: The thickness of accumulated blood is less than 1 mm, and no blood clot is formed. Grade III: Hematocele thickness>1 mm. Grade IV: There is obvious hematocele or intracerebral hematoma in the cerebral ventricle. ^(2)^Hunt-Hess grade: Grade I: No obvious symptoms, with or without mild headache. Grade II: Obvious headache, oculomotor nerve paralysis, stiff neck. Grade III: Slight disturbance of consciousness. Grade IV: In a state of hemiplegia and semi coma. Level V: Deep coma.

Factors	Good prognosis group<br>(n=151)	Poor prognosis group<br>(n=49)	χ^2^	P
Age			2.213	0.137
≤60 years old	77 (50.99)	19 (38.78)		
>60 years old	74 (49.01)	30 (61.22)		
Gender			2.457	0.117
Male	78 (51.66)	19 (38.78)		
Female	73 (48.34)	30 (61.22)		
Surgery timing			6.611	0.010
Early	99 (65.56)	22 (44.90)		
Delayed	52 (34.44)	27 (55.10)		
Aneurysm location			5.473	0.019
Anterior circulation	102 (67.55)	24 (48.98)		
Posterior circulation	49 (32.45)	25 (51.02)		
Aneurysm diameter			0.791	0.374
≤15 mm	94 (62.25)	27 (55.10)		
>15 mm	57 (37.75)	22 (44.90)		
Tumor neck width			11.114	0.001
≤4.5 mm	102 (67.55)	20 (40.82)		
>4.5 mm	49 (32.45)	29 (59.18)		
Fisher grade^(1)^			9.808	0.002
I~II	113 (74.83)	25 (51.02)		
III~IV	38 (25.17)	24 (48.98)		
Hunt-Hess grade^(2)^			8.014	0.005
I~II	120 (79.47)	29 (59.18)		
III~IV	31 (20.53)	20 (40.82)		
Hypertension			7.677	0.006
Yes	34 (22.52)	21 (42.86)		
No	117 (77.48)	28 (57.14)		
Diabetes			0.407	0.523
Yes	22 (14.57)	9 (18.37)		
No	129 (85.43)	40 (81.63)		
Hyperlipidemia			0.004	0.947
Yes	21 (13.91)	7 (14.29)		
No	130 (86.09)	42 (87.71)		
Smoking history			1.217	0.270
Yes	36 (23.84)	8 (16.33)		
No	115 (76.16)	41 (83.67)		

### Multifactorial logistic regression model analysis of prognostic influencing factors

Multifactorial analysis showed that delayed surgery, aneurysm in the posterior circulation, aneurysm neck width >4.5 mm, Fisher grade III-IV, Hunt-Hess grade III-IV, and hypertension were all independent risk factors for poor prognosis after IA interventional embolization (P<0.05) (Table 4-Table 5).

**Table 4:** Multifactorial logistic regression model analysis of prognostic influencing factors.

Factors	Variable	Assignment
Surgery timing	X1	early surgery=0, delayed surgery=1
Aneurysm location	X2	anterior circulation=0, posterior circulation=1
Tumor neck width	X3	≤4.5 mm=0, >4.5 mm=1
Fisher grade	X4	I=0, II=2, III=3, IV=4
Hunt-Hess grade	X5	I=0, II=2, III=3, IV=4
Hypertension	X6	yes=0, no=1

**Table 5:** Assignment table. Note: A multivariate logistic regression model analyzed the prognostic factors.

Factors	β	SE	Wald	P	OR	95%CI
Delayed surgery	0.670	0.332	4.075	0.041	1.954	1.019~3.746
Aneurysm in posterior circulation	0.652	0.318	4.034	0.037	1.919	1.029~3.580
Tumor neck width > 4.5 mm	0.933	0.240	7.524	0.004	2.542	1.588~4.069
Fisher grade III~IV	1.127	0.475	10.350	0.000	3.086	1.217~7.830
Hunt-Hess grade III~IV	1.085	0.431	9.652	0.000	2.959	1.272~6.888
Hypertension	1.204	0.402	12.647	0.000	3.333	1.516~7.330

## Discussion

IA is a cerebrovascular disease characterized by an abnormal aneurysm of the wall of the intracranial artery. Once the tumor ruptures and bleeds, the patients may become disabled or even die. Data [15] show that more than 25% of patients with IA rupture died without timely and effective treatment, and at least half of the survivors were associated with severe neurological deficits, which is extremely detrimental to patient safety and quality of life. As a minimally invasive procedure, EVET places the aneurysm in the blood circulation, and the procedure is not affected by hydrocephalus and intracranial pressure, which is safer and less traumatic, can effectively reduce the surgical trauma, avoid mechanical damage or stimulation of tissues and blood vessels around the aneurysm capsule, and improve the postoperative recovery [16]. However, the choice of the timing of surgery is controversial and this study analyses this and examines its prognostic factors.

In our results, the early group is superior to the delayed group in improving the effect of intraoperative embolization and promoting postoperative neurological recovery. This may be because although patients are prone to cerebral vasospasm in the early stage of IA are prone to cerebral vasospasm, the interventional technology is more mature now, and the obstruction of micro catheter implantation into the aneurysm is smaller, which will not affect the embolization effect. On the contrary, in patients with delayed surgery, with the prolongation of the IA attack, the ischemic brain injury becomes more severe, and the risk of tumor rupture and bleeding increases. If the tumor rupture and bleeding exceed 72 h, the red blood cells will be completely dissolved, and the difficulty of filling the coil and implanting the micro catheter will be greatly increased, which will promote the patients' thrombosis and also increase the risk of cerebral vasospasm. Therefore, it is not conducive to the realization of the surgical effect and the recovery of neurological function after surgery [17].

The important role of serum MMP-9 in vivo is to decompose the extracellular matrix, destroy the integrity of cerebral blood vessels, and have a certain influence on matrix remodeling [9]. Zou et al. [18] found that the abnormal increase of serum MMP-9 levels in IA patients plays a role in destroying the blood-brain barrier and aggravating brain tissue damage, which is an important factor affecting the prognosis of patients. El Shimy et al. [19] showed that the levels of NSE was closely related to the degree of brain tissue injury and prognosis of IA patients. When brain tissue is damaged, ischemic or edematous, NSE can enter the blood through the blood-brain barrier, which makes the level of NSE in the blood increase obviously, suggesting that the prognosis of patients can be evaluated according to the level of serum NSE in clinic to guide clinical treatment [10]. MPO is a serum inflammatory factor involved in tissue cell damage at the site of inflammation and is associated with the formation, development, and rupture of aneurysms [11]. During vasospasm, the level of MPO in blood of IA patients increased, suggesting that MPO can promote the formation, development and rupture of IA. In the current results, the serum levels of MMP-9, NSE, and MPO in the early group were significantly lower than those in the delayed group after surgery, and the good prognosis rate in the early group was higher than that in the delayed group. This indicates that the early intervention treatment of IA can improve the prognosis of patients, protect brain tissue and prevent aneurysm rupture and bleeding by reducing the inflammation of arterial wall.

In addition to the above, the multivariate analysis in this study found that posterior circulation aneurysm, aneurysm neck width >4.5 mm, Fisher grade III-IV, Hunt-Hess grade III-IV, and hypertension were independent risk factors for poor prognosis after IA interventional embolization. The reasons for these results are analyzed: the aneurysms of the posterior circulation are deeper, most of the aneurysms are more complicated, especially the large arteries and wide-necked aneurysms, which are more severe and have a higher mortality, whereas the aneurysms of anterior circulation are relatively more regular in shape, with a lower surgical risk and a better prognosis. The width of the aneurysm neck affects the speed of blood flow velocity into the aneurysm. The greater the width, the faster the flow rate, and the greater the pressure, which are more likely to cause aneurysm rupture and lead to poor prognosis. Fisher grade is related to whether there is subarachnoid hemorrhage. The more serious the hemorrhage, the worse the surgical prognosis [20]. Hunt-Hess grade is related to nerve function, and the higher grade indicates more nerve function damage and higher mortality [21]. Persistent high blood pressure levels can cause atherosclerosis of the arterial wall, leading to decreased elastic fiber function and vessel wall degeneration, which may induce aneurysm rupture, cerebral infarction, and cerebral ischemia, affecting patient prognosis [22].

In this study, we discussed the clinical effects of different treatment opportunities of EVET in IA, and found that early interventional therapy is more effective, which has certain reference value for choosing treatment opportunities and improving patients' prognosis. The study also observed the changes of serum MMP-9, NSE and MPO concentrations in patients with IA and the related factors affecting the prognosis of patients, which is helpful for postoperative monitoring and clinical prevention and treatment of patients with IA. However, there are still some limitations in the study. Firstly, the number of cases is small, and it is necessary to accumulate cases in the follow-up study to support the conclusion. Secondly, the specific mechanism of MMP-9, NSE and MPO regulating the prognosis of IA patients is still unclear, which needs further study.

## Conclusion

Early interventional embolization for IA patients can improve their complete embolization rate and reduce serum MMP-9, NSE, and MPO levels; delayed surgery, aneurysm in posterior circulation, aneurysm neck width >4.5 mm, Fisher grade III-IV, Hunt-Hess grade III-IV, and hypertension are closely associated with poor prognosis after interventional embolization in IA patients.

## Dodatak

### Data Availability

The data to support the results of the study are available on request from the corresponding author.

### Funding

It's supported by Sichuan science and technology department (2021YFS0082) and Chengdu Science and Technology Bureau (2021-YF05-02242-SN).

### Conflict of interest statement

All the authors declare that they have no conflict of interest in this work.

## References

[1] Dietrich P (2020). Influence of genetics in intracranial aneurysms. Radiologe.

[2] Tang H, Wang Q, Xu F, Zhang X, Zeng Z, Yan Y, et al (2021). Underlying mechanism of hemodynamics and intracranial aneurysm. Chin Neurosurg J.

[3] Wen D, Wang X, Chen R, Li H, Zheng J, Fu W, et al (2022). Single-Cell RNA Sequencing Reveals the Pathogenic Relevance of Intracranial Atherosclerosis in Blood BlisterLike Aneurysms. Front Immunol.

[4] Signorelli F, Sela S, Gesualdo L, Chevrel S, Tollet F, Pailler-Mattei C, et al (2018). Hemodynamic Stress, Inflammation, and Intracranial Aneurysm Development and Rupture: A Systematic Review. World Neurosurg.

[5] Leao D J, Agarwal A, Mohan S, Bathla G (2020). Intracranial vessel wall imaging: Applications, interpretation, and pitfalls. Clin Radiol.

[6] Rinkel G J, Ruigrok Y M (2022). Preventive screening for intracranial aneurysms. Int J Stroke.

[7] Sonig A, Shallwani H, Natarajan S K, Shakir H J, Hopkins L N, Snyder K V, et al (2018). Better Outcomes and Reduced Hospitalization Cost are Associated with Ultra-Early Treatment of Ruptured Intracranial Aneurysms: A US Nationwide Data Sample Study. Neurosurgery.

[8] Stephan S, Blanc R, Zmuda M, Vignal C, Barral M, Pistocchi S, et al (2016). Endovascular treatment of carotid-cavernous fistulae: Long-term efficacy and prognostic factors. J Fr Ophtalmol.

[9] Johnson A K, Munich S A, Tan L A, Heiferman D M, Keigher K M, Lopes D K (2015). Complication analysis in nitinol stent-assisted embolization of 486 intracranial aneurysms. J Neurosurg.

[10] Wang W, Guo Z, Xie D, Lin Z, Lin R (2022). Relationship between MMP-9 Gene Polymorphism and Intracranial Aneurysm. Cell Mol Biol.

[11] Zheng D, Zhao S, Zhang N, Shi J (2020). Brain protective effect and hemodynamics of dexmedetomidine hydrochloride in patients with intracranial aneurysm. Saudi J Biol Sci.

[12] Zhang Z, Sui R, Ge L, Xia D (2021). CircRNA_0079586 and circRNA_RanGAP1 are involved in the pathogenesis of intracranial aneurysms rupture by regulating the expression of MPO. Sci Rep-Uk.

[13] Steiner T, Juvela S, Unterberg A, Jung C, Forsting M, Rinkel G (2013). European Stroke Organization guidelines forthe management of intracranial aneurysms and subarachnoid haemorrhage. Cerebrovasc Dis.

[14] 14. Neurointerventional Group of the Chinese Medical Association Neurosurgery Branch (2013). Chinese expert consensus on endovascular intervention for intracranial aneurysms (2013). Chin Med J.

[15] Dorsch N (2011). A clinical review of cerebral vasospasm and delayed ischaemia following aneurysm rupture. Acta Neurochir Suppl.

[16] Li H, Gao B L, Li C H, Wang J W, Liu J F, Yang S T (2020). Endovascular Retreatment of Cerebral Aneurysms Previously Treated with Endovascular Embolization. J Neurol Surg Part a.

[17] Jiang C, Luan D, Wang C, Liu Q, Han J, Li G (2019). Risk and Prognostic Factors for Rupture of Intracranial Aneurysms During Endovascular Embolization. World Neurosurg.

[18] Zou L, Hou Y, Yu B, Li S, Du Y (2018). The effect of intravascular interventional embolization and craniotomy on MMP-2, MMP-9 and caspase3 in serum of intracranial aneurysm patients. Exp Ther Med.

[19] El S M, El-Raggal N M, El-Farrash R A, Shaaban H A, Mohamed H E, Barakat N M, et al (2018). Cerebral blood flow and serum neuron-specific enolase in early-onset neonatal sepsis. Pediatr Res.

[20] Ridwan S, Urbach H, Greschus S, Hagen J V, Esche J, Bostrom A (2021). Health Economic Aspects of Aneurysmal Subarachnoid Hemorrhage: Factors Determining First Year In-Hospital Treatment Expenses. J Neurol Surg Part a.

[21] Mahta A, Murray K, Reznik M E, Thompson B B, Wendell L C, Furie K L (2021). Early Neurological Changes and Interpretation of Clinical Grades in Aneurysmal Subarachnoid Hemorrhage. J Stroke Cerebrovasc.

[22] Gutierrez J, Turan T N, Hoh B L, Chimowitz M I (2022). Intracranial atherosclerotic stenosis: Risk factors, diagnosis, and treatment. Lancet Neurol.

